# Osseointegration of Implants Through Ti Biofunctionalization with Biomass from *Chlorella sorokiniana* UTEX 1230 and *Synechococcus* sp. PCC 7002

**DOI:** 10.3390/ijms252313161

**Published:** 2024-12-07

**Authors:** Yarelis Bravo, Alejandra M. Miranda, Fabian Hernandez-Tenorio, Alex A. Sáez, Virginia Paredes

**Affiliations:** 1 Biological Sciences and Bioprocesses Group, School of Applied Sciences and Engineering, Universidad EAFIT, Medellín 050022, Colombia; ybravor@eafit.edu.co (Y.B.); ammirandap@eafit.edu.co (A.M.M.); asaez@eafit.edu.co (A.A.S.); 2 Environmental Processes Research Group, School of Applied Sciences and Engineering, Universidad EAFIT, Medellín 050022, Colombia; fehernandt@eafit.edu.co; 3 Biomedical Engineering Department, Universidad Simón Bolívar, Barranquilla 080002, Colombia

**Keywords:** microalgae, proteins, titanium, biomass, osseointegration, orthopedic, biofunctionalization

## Abstract

The inadequate osseointegration of titanium implants remains a significant challenge in orthopedics, limiting the long-term efficacy of prostheses and medical devices. It has been determined that biological aging of the titanium surface compromises the implant–bone tissue interaction due to increased hydrophobicity and accumulation of organic molecules. To address this issue, an innovative strategy has been proposed: the biofunctionalization of Ti6Al4V surfaces utilizing biomass derived from *Chlorella sorokiniana* UTEX 1230 and *Synechococcus* sp. PCC 7002. This research was structured to encompass microalgal culture optimization through biocompatibility evaluation of biofunctionalized surfaces. Biofunctionalization stages were analyzed using contact angle measurements, EDS, SEM, and cellular assays. It was observed that piranha solution activation generated a hydrophilic surface, while silanization was more efficient in samples treated for 14 h. It was found that *Synechococcus* sp. PCC 7002 presented a higher biomass concentration on the surface compared to *C. sorokiniana* UTEX 1230. Cytotoxicity assays revealed that the coating with *Synechococcus* sp. PCC 7002 was potentially non-cytotoxic, with a cell viability of 86.8%. SEM images showed a significant number of cells adhered to the treated sample. In conclusion, the potential of using microalgal biomass to biofunctionalize titanium surfaces has been demonstrated, offering an innovative alternative to improve implant–tissue interaction and, consequently, the osseointegration process in orthopedic applications.

## 1. Introduction

Titanium (Ti) and some of its alloys have been widely used as orthopedic implants due to their mechanical properties, corrosion resistance, wear resistance, inert nature, ability to adsorb proteins on their surface, and excellent biocompatibility [1,2,3]. However, titanium undergoes biological aging characterized by increased hydrophobicity and surface accumulation of organic molecules over time, compromising osseointegration [4,5]. Researchers have demonstrated that such surface modifications can significantly improve implant performance by providing a more suitable environment for protein adsorption and cell growth, thereby enhancing overall biocompatibility and integration with surrounding tissues [6,7]. One strategy to achieve these multicomponent surfaces is using biomolecules in a biofunctionalization process [2]. This process involves modifying the physicochemical properties of the material by inserting functional groups that facilitate the incorporation of other molecules or biomolecules, aiming to enhance the biological response of the organism to the implant material [7]. Rappe et al. [2] and Park et al. [8] employed synthetic biomolecules, such as proteins, peptide sequences, lipids, nucleic acids, carbohydrates, and polysaccharides, for the surface immobilization of biomaterials, thereby improving their biological interaction [3].

Recent advances in biomedicine focus on the immobilization of biomolecules, such as proteins, on titanium surfaces. These biomolecules act as mediators of cell adhesion and as growth and proliferation stimulants for tissue cells, as these molecules are naturally present in the tissues that the device must replace [1,9]. Additionally, due to their biological origin, these molecules are not recognized as foreign agents, thus preventing immune rejection of the prosthesis [2,10].

Among the microorganisms used to produce single-cell proteins (SCPs), microalgae have the highest protein content (60–70% *w*/*w*), followed by bacteria (30–80% *w*/*w*), yeasts (30–50% *w*/*w*), and protists (10–20% *w*/*w*) [11,12]. In addition to using CO_2_, light, and nutrients for biomass production, microalgae and cyanobacteria have the capability to produce economically valuable chemical compounds (Richmond, 2013 [13]) such as carotenoids, proteins, carbohydrates, lipids, essential amino acids, vitamins, minerals, and polyunsaturated fatty acids [14]. Among these microorganisms is *Chlorella sorokiniana* UTEX 1230, characterized by its rapid growth rate [14] and high content of lipids (11–31%), carbohydrates (11–55%), proteins (24–45%), inorganic minerals (1–2%), antioxidants (4%), and vitamins (0.12%) in its structure [13,15]. On the other hand, *Synechococcus* sp. PCC 7002 is a euryhaline unicellular cyanobacterium with several attractive features for biological research as well as biotechnological and industrial applications [8], including high protein content (53%) and carbohydrates (40%) [16].

The use of these microorganisms to biofunctionalize Ti surfaces offers several significant advantages, especially in biomedical applications. These include enhanced biocompatibility, natural antimicrobial properties that reduce the risk of infections in implants, and improved stability and durability of the implant [17]. Therefore, the aim of the present study was to biofunctionalize the Ti surface with microalgal biomass from strains 1230 and 7002 to improve the osseointegration of titanium prostheses.

## 2. Results and Discussion

### 2.1. Biomass Production of 1230 and 7002

**Figure 1:**
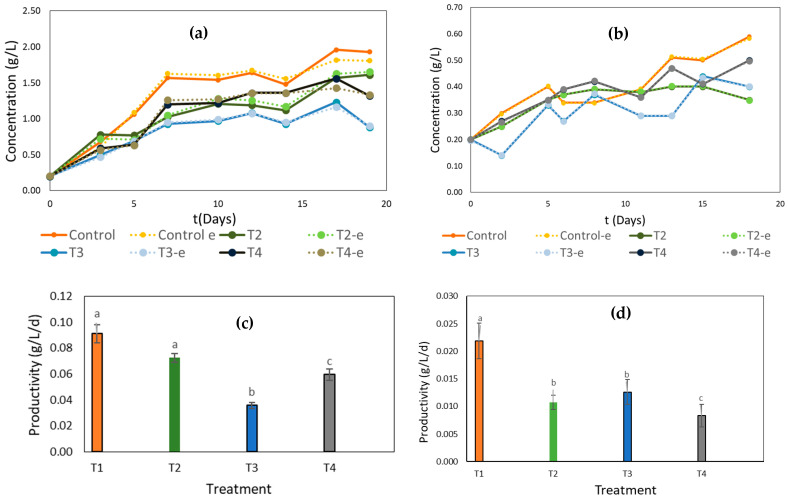
Growth kinetics under different nitrogen source concentrations of (**a**) 1230, (**b**) 7002, and biomass productivity of (**c**) 1230 and (**d**) 7002. Identical letters correspond to statistically homogeneous groups according to Tukey’s test, with a 95% confidence level.

The growth kinetics of strain 1230 conformed to the logistic model (R^2^ > 0.98), similar to strain 7002 (R^2^ > 0.99), as illustrated in Figure 1a,b. This indicates that the experimental values were closely aligned with the predicted values of the model, as reported by Bhushan et al. [18] and Padil et al. [19] for the growth kinetics of *Chlorella sorokiniana*, *C*. *vulgaris*, and *Scenedesmus obliquus*. Both strains thrived in various treatments of the BBM medium and fertilizers, under different concentrations of the nitrogen source, and their growth phases were presented in a similar way in the different treatments.

For strain 1230, an adaptation phase of one day was observed, followed by an exponential phase that concluded between the tenth and seventeenth days of cultivation. Conversely, strain 7002 exhibited a similar adaptation phase, with the exponential phase ending between the thirteenth and eighteenth days. Regarding productivity, the highest value was achieved with treatment T1 for both strains, corresponding to the BBM medium. The lowest productivity for strain 1230 was observed with treatment T3, while for strain 7002, it was treatment T2, showing a significant difference (*p* < 0.05) between the average values, as shown in Figure 1c,d. This is possibly attributed to the critical role of nitrogen in cell division, where an excess of nutrients can inhibit microalgal growth [20]. Thus, higher concentrations of this nutrient do not necessarily lead to increased nitrogen consumption or biomass production.

The interaction effect on protein accumulation for both strains is presented in Table 1. For strain 1230, the treatment with the highest protein percentage was T3, with a value of 47.70%, corresponding to the use of synthetic fertilizer as the medium. This suggests that a lower cell concentration allows for greater resource availability per cell for metabolite production, prioritizing protein synthesis. Similar findings were reported by Safafar et al. [21] and Xie et al. [22], who evaluated this factor in the cultivation of *Chlorella pyrenoidosa* and *Chlorella vulgaris*, achieving protein contents of 65.2% and 44.3%, respectively. On the other hand, for strain 7002, the highest protein production was observed in treatment T1, with 48.14%, corresponding to Zarrouk’s medium. This indicates that a higher nitrogen concentration in the medium does not ensure a high protein content in the resultant biomass, as noted by Wang et al. [23], when cultivating various species, such as *Chlorella vulgaris*, *Desmodesmus* sp., and *Scenedesmus obliquus*, with nitrogen concentrations ranging from 0.5 to 1.0 g/L, resulting in an increase in protein content from 38% to 46%, and with no further increases observed when nitrogen concentration exceeded 1.0 g/L. This is likely due to the nitrogen content being sufficient for cell division and protein synthesis.

The biomass obtained from these two treatments was used for subsequent stages. A proximate analysis was conducted (Table 2), revealing minor differences among the other measured parameters. This confirmed that nitrogen represents a critical macronutrient regulating metabolism and, consequently, the growth rate and biochemical composition of microalgae, including the synthesis of proteins, lipids, and carbohydrates [23].

### 2.2. Activation

**Figure 2:**
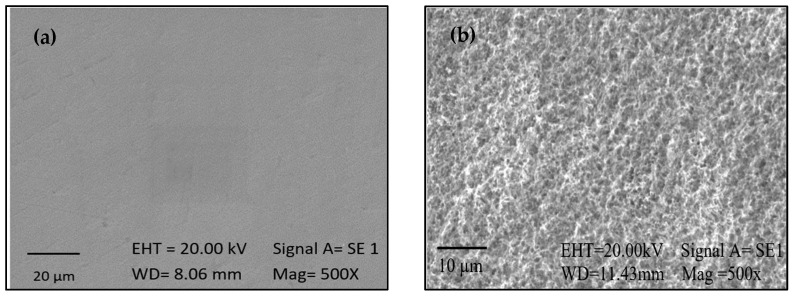
SEM images of the surface of (**a**) control Ti and (**b**) Ti activated with a 1:1 piranha solution.

The contact angle evaluation of the control sample, an untreated specimen, exhibited an angle of 76°, while the treated sample demonstrated an angle of 18°. The enhancement in hydrophilicity is closely associated with the cleaning of the surface or the elimination of contaminants [24]. The results suggest that the piranha solution promoted a more hydrophilic surface. The reduction in the contact angle can likely be attributed to the incorporation of hydroxyl groups on the surface, resulting from the reaction between sulfuric acid and hydrogen peroxide. Chouirfa et al. [25] stated that among the techniques for titanium oxidation, the combination of sulfuric acid and peroxide is one of the most effective, as it provides a more uniform surface with fewer impurities. Furthermore, the oxide layer is essential for coupling with silanes, as it offers a surface rich in hydroxyl groups, enhancing the reactivity of silanol groups and thus facilitating the formation of strong covalent bonds [26,27]. Additionally, SEM micrographs of the activation stage are presented in Figure 2. A modification in the surface was observed, transitioning from a uniform, polished surface (Figure 2a) to one with irregularities and increased roughness due to chemical etching (Figure 2b). Similar results were reported by Mallaiah et al. [28], in their review, which presents the outcomes of surfaces after various activation techniques, including chemical etching.

### 2.3. Silanization

Surface modification is also employed to enhance osseointegration, achieve faster healing times, improve bone implant contact, and increase the lifespan of titanium implants [25,28]. Silanization has been successfully used to functionalize metallic biomaterials with bioactive molecules. This surface modification method allows for the covalent bonding of various molecules, such as peptides, proteins, and polymers, using organofunctional alkoxysilane molecules that react with hydroxyl groups present on the surface of material. Senna et al. [29] affirmed that this modification did not affect the osseointegration process. Other researchers, such as Chen et al. [30], have reported on the anchoring of silane for the grafting of melimine, a synthetic antimicrobial peptide, onto titanium surfaces. Another instance of silanized titanium coating was described by Hasan et al. [20], who conducted a study on the effects of Ti6Al4V surface functionalization on protein adsorption and cellular behavior. In relation to the biofunctionalization of metallic surfaces.

**Figure 3:**
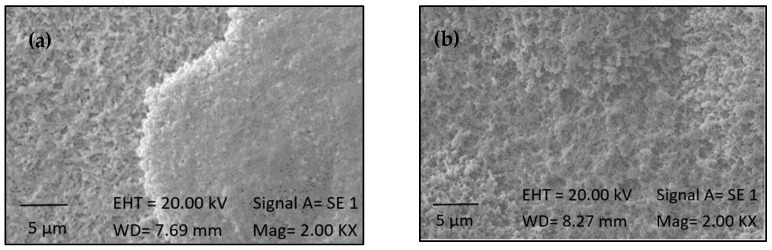
SEM images of samples after the silanization process, with durations of (**a**) 3 h and (**b**) 14 h.

Figure 3 presents images of the titanium samples subjected to the silanization process for two different durations. Morphological modifications are evident in both cases. The rough film observed in white and gray tones is inferred to be the silane layer adhered to the substrate. Additionally, a difference in surface morphology is observed between the two silanization durations, with the sample treated for 14 h (Figure 3b) exhibiting greater porosity and roughness compared to the sample treated for 3 h (Figure 3a), and according to [31], these changes in topology improve osseointegration due to the absorption of proteins, especially collagen, on the surface.

Table 3 presents the EDS analysis results for the control samples and those subjected to silanization for two treatment durations. Regarding the control and in accordance with the chemical composition of the sample used, the results obtained are as expected [8], as only the elements present in this alloy (Ti6Al4V) were recorded. With respect to the silanized specimens, the presence of silicon and carbon on their surfaces was detected because of treatment with 3-aminopropyltriethoxysilane (APTES), which are the primary components of the silane film. APTES forms covalent Si–O–Metal bonds on the TiAlV surface, which can lead to a measurable decrease in the metallic elements signal during EDS analysis due to the overlay of the silane layer. A statistically significant difference (*p* < 0.05) in the content of these elements was observed, with the specimen treated for 14 h showing a higher percentage of these atoms on its surface compared to those treated for 3 h [32].

The silanization process was demonstrated to be effective in inducing physical and chemical modifications on activated surfaces, thereby confirming the formation of the silane layer. Observations indicated more favorable outcomes for samples treated for 14 h, leading to their selection for subsequent phases. A surface with increased roughness is considered ideal for immobilization processes. The irregularities and pores on a rough surface provide mechanical anchorage and expose reactive functional groups, facilitating the formation of covalent bonds. This enhances the retention and stability of immobilized molecules, which is crucial for medical implant applications because to improve osseointegration [27,33].

### 2.4. Immobilization of Biomass from 1230 and 7002

A wide array of biological molecules has been investigated to develop bioactive surfaces capable of enhancing bone generation and the osseointegration of titanium implants. For example, Sánchez-Bodón et al. [34] reported bioactive coatings on titanium, highlighting various materials such as antimicrobial peptides, polymers, antimicrobial and antifungal agents, and hydroxyapatite. Similarly, other researchers, such as Gurzawska et al. (2012) [12], utilized polysaccharides as coatings, while Park et al. (2021) [8] employed proteins and glycoproteins to biofunctionalize titanium. Additionally, Kashin et al. [35] utilized diatomite-based coatings, a biogenic material composed of microalgae and diatom shells. Gutierrez-Pua et al. [36] affirmed that biofunctionalization of magnesium surfaces with dicalcium phosphate dihydrate (DCPD) and *Chlorella* sp. biomass presents a promising approach to enhance corrosion resistance for potential orthopedic applications. In a similar manner, in this study, biomass from a microalga (1230) and a cyanobacterium (7002) was used as a coating.

**Figure 4:**
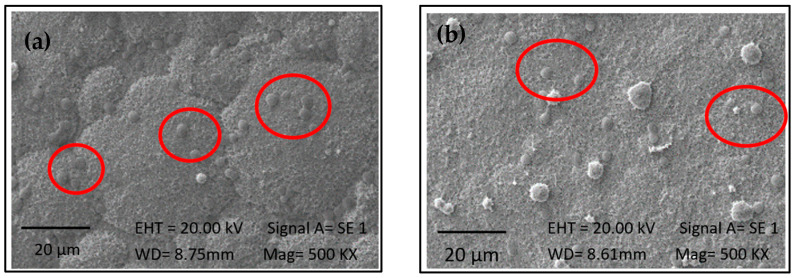

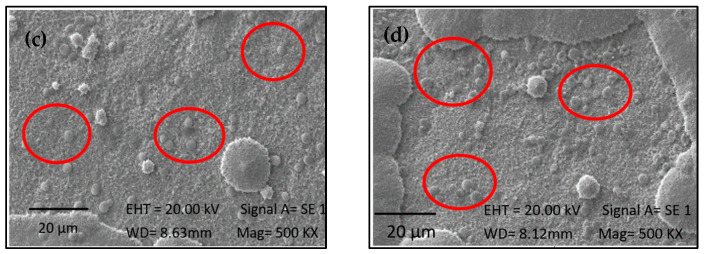
SEM images of samples with immobilized biomass of 1230: (**a**) at 3 g/L for 14 h, (**b**) at 5 g/L for 3 h, (**c**) at 5 g/L for 14 h, and (**d**) at 5 g/L for 14 h. The red circle highlights the spherical shape of the strain.

*Chlorella* is characterized by spherical cells with a reported size ranging from 3 to 8 µm in diameter. As a result of this study, microalgae with an average size of 8 µm were obtained (UTEX), as depicted in Figure 4 and highlighted in red circles. For samples treated at a concentration of 3 g/L, no significant differences were observed between the two evaluated times (*p* < 0.05). Conversely, at a concentration of 5 g/L, the sample immersed for a longer duration in the biomass exhibited a greater cell population on the surface. It can be affirmed that higher biomass concentration and exposure time increase the concentration of microalgae on the material surface. This is attributed to the availability of more molecules to interact with the surface, increasing the likelihood of encountering binding sites and facilitating covalent bond formation, thereby enhancing adhesion probabilities. Felgueiras et al. [37] reported that a higher substrate concentration on a surface typically leads to greater adhesion. However, once all binding sites on the surface are occupied, a saturation point is reached where adhesion does not significantly increase with higher concentrations.

The chemical composition of the synthesized coatings was characterized using the energy-dispersive X-ray microanalysis method. The literature indicates a higher presence of nitrogen in samples coated with biomass, which is consistent with the EDS results for sample 1230. The EDS results for sample 1230, as presented in Table 4, which showed a decrease in the percentage of metallic elements compared to the silanized samples (Table 3), were attributed to the overlay of the microalgae layer. This overlay is further evidenced by the presence of oxygen, phosphorus, and nitrogen as well as an increased carbon content. The incorporation of these elements, along with hydrogen, within the microalgae structure provides strong evidence of successful biomass immobilization on the substrate. The presence of these components, characteristic of microalgal composition, confirms that the coating process effectively resulted in the attachment of microalgal biomass to the implant surface [9].

**Figure 5:**
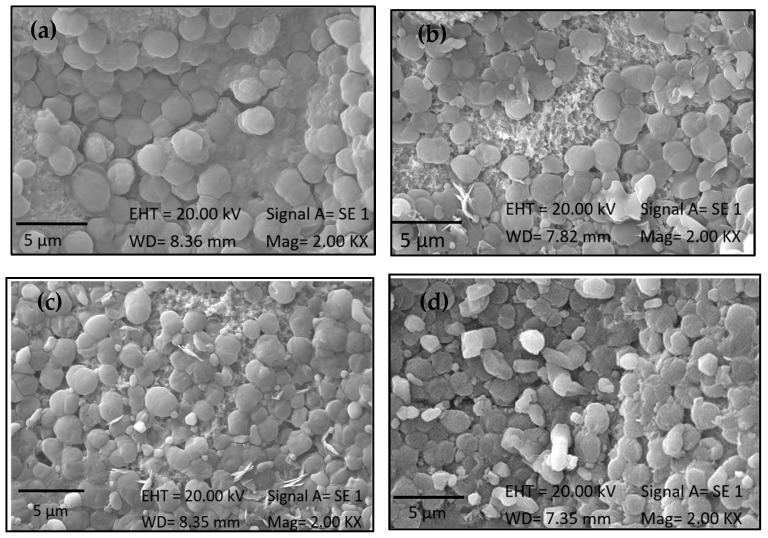
SEM images of samples after immobilization of *Synechococcus* sp. PCC 7002 biomass: (**a**) at 3 g/L for 3 h, (**b**) at 5 g/L for 3 h, (**c**) at 3 g/L for 14 h, and (**d**) at 5 g/L for 14 h.

In contrast, *Synechococcus* is characterized by spherical cells (0.8–1.5 µm in diameter) [38], which were observed in the images as small circles distributed across the surface, as shown in Figure 5. For the treated samples, no significant difference was observed between treatments at both concentrations (3 g/L and 5 g/L), with a substantial amount present on the surface. However, for the 5 g/L concentration with a duration of 14 h (Figure 5d), the defined shape of the microalgae was not discernible, suggesting that a high quantity of microalgae over an extended period occupied all available binding sites, leading to overlapping [37]. It should be noted that these results do not interfere with subsequent tests; thus, it can be stated that a higher concentration of biomass and a longer exposure time increase the effectiveness of microalgae adhesion to the surface of the material because of the availability of more molecules to interact with the surface, increasing the likelihood of encountering binding sites, facilitating covalent bond formation, and thereby enhancing the adhesion probability.

The EDS results for sample 7002 following biomass immobilization demonstrated the presence of oxygen, phosphorus, and nitrogen, as shown in Table 5. Additionally, the carbon content increased compared to the silanized samples (Table 3). The presence of these elements is attributed to their integration into the cyanobacteria structure, confirming biomass immobilization [39].

### 2.5. Effectiveness of Immobilization

**Figure 6:**
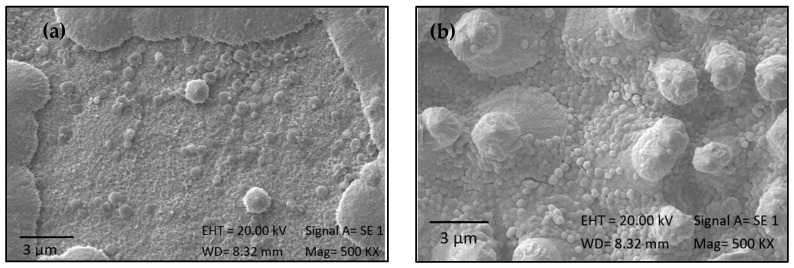
SEM images of samples after biomass immobilization: (**a**) 1230 at 5 g/L for 14 h and (**b**) of 7002 at 5 g/L for 14 h.

**Figure 7:**
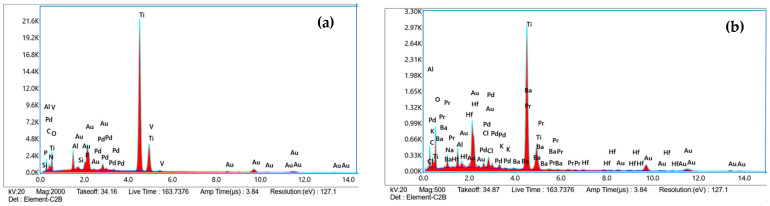
EDS spectra of samples after biomass immobilization: (**a**) 1230 at 5 g/L for 14 h and (**b**) of 7002 at 5 g/L for 14 h.

A significant difference in the concentration of microalgae on the surface was observed when comparing the most favorable results from both microalgae strains (*p* < 0.05). According to the SEM micrographs shown in Figure 6, a greater presence of cells was observed on the surface treated with strain 7002 (b) compared to the surface treated with strain 1230 (a). Furthermore, EDS spectra confirm the presence of elements that form microalgae structures, such as carbon, oxygen, nitrogen, and phosphorus (Figure 7a,b). This is attributed to the influence of microalgae size on their immobilization on the titanium surface. Smaller particles possess a larger specific surface area compared to larger particles [40], allowing them to establish more physical interactions, such as Van der Waals forces, and chemical interactions, such as covalent or ionic bonds, with the surface [33]. Additionally, these smaller particles can better penetrate surface irregularities and pores, thereby increasing the effective contact area and enhancing adhesion [41]. It has been demonstrated that nanoparticles yield better results in coating materials, as reported by Gurzawska et al. [12], who demonstrated in an in vitro study that several polysaccharide nanocoatings exhibited increased cell adhesion, proliferation, and mineralization. The authors asserted that these coatings affected both surface properties and cellular reactions, potentially improving osseointegration.

### 2.6. Cellular Assays

For the cellular assays, the most effective treatment identified in previous stages was selected. Cytotoxicity, viability, and cell adhesion assays were conducted on the surface treated with strain 7002, at a concentration of 5 g/L and an immobilization time of 14 h. These assays were performed using osteoblastic cells, as they play a fundamental role in osseointegration, being responsible for synthesizing and mineralizing the bone matrix [42]. This approach also explains the behavior of these biofilms when in contact with human tissue.

#### 2.6.1. Cytotoxicity and Cell Viability

Cytotoxicity assays are commonly employed to evaluate the damage or loss of cellular or intracellular components and functions, including lethal cytotoxicity levels, offering a clear indication of their potential to cause harm to cells or tissues [43]. Cell viability was assessed using the MTT assay. After a 48-h incubation period with Saos-2 cells. It was found that, compared to the control, which was only silanized, cells treated with the 7002 coating did not exhibit a significant decrease in viability (*p* > 0.05); the viability was approximately 88.9% and 86.8%, respectively (Table 6). These values are comparable to those observed in untreated control cells, meaning those that were not silanized, indicating that the treatments did not affect cell viability and can thus be classified as potentially non-cytotoxic coatings [44].

#### 2.6.2. Cell Adhesion

A novel strategy to enhance implant integration is the development of surface coatings that increase bone cell adhesion, a crucial factor in the biocompatibility of materials, especially at their contact surfaces [45,46]. Biocompatibility refers to the ability of a material to support cell adhesion on its surface [12]. Additionally, it has been shown that cell adhesion is intrinsically linked to appropriate surface morphology. Authors such as Jayaraman et al. [47] and Zhu et al. [46] concluded that increasing the material’s roughness enhances the contact area between the implant and tissue, resulting in greater cell adhesion.

**Figure 8:**
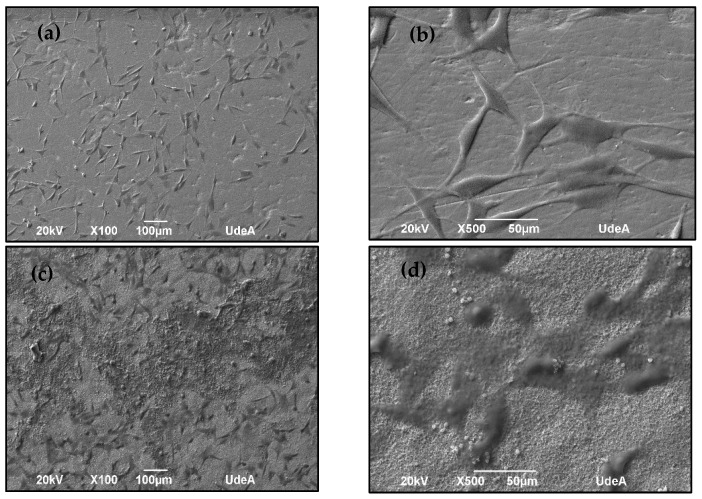
SEM micrographs of the cell adhesion assay on the titanium surface: (**a**) untreated at 100X, (**b**) untreated at 500X, (**c**) coated with biomass of strain 7002 at 100X, and (**d**) coated with biomass of strain 7002 at 500X.

When evaluating the capacity of the microalgae-coated surface to adhere Saos-2 cells, variation was observed compared to the untreated surface. The adhesion assay demonstrated the presence of osteoblasts adhered to the material surfaces, as shown in Figure 8. After 24 h of incubation, SEM microscopy revealed cells adhered to the treated sample, displaying a polyhedral morphology characteristic of this cell type and the formation of a cellular monolayer. No alterations, such as fragmentation indicating stress or cell death, were detected. These results allowed the behavior of these coatings to be assessed in relation to human tissue, showing potential improvement in osseointegration. This is promising, considering that these cells adhere to the surface through at least two different mechanisms: direct adhesion, where bone cells adhere directly to the surface, and indirect adhesion, resulting in the binding of various proteins from surrounding fluids to the surface [12]. These proteins selectively bind to different receptors on the surface of invading cells. Receptor binding triggers signals in the cells, resulting in increased adhesion, proliferation, and extracellular matrix production [48].

## 3. Materials and Methods

### 3.1. Materials

Commercial Ti6Al4V alloy discs, each with a diameter of 8 mm and a thickness of 7 mm, were utilized. Prior to biofunctionalization, the samples were polished using SiC paper with grit sizes of 400, 600, 1000, and 1200. This was followed by ultrasonic cleaning (Elma Ultrasonic LC30 H, Singen, Germany) for 3 min in each of the following solvents: water, 90% *v*/*v* ethanol, and acetone (PanReac AppliChem, Darmstadt, Germany).

### 3.2. Activation and Silanization

The modification of the surface was initiated with an activation process aimed at generating hydroxyl groups (OH-) on the surface of the material to improve surface disposition for subsequent treatments, such as silanization or molecule adhesion [36]. This was achieved through a piranha solution reaction (H_2_SO_4_ 98% *v*/*v* and H_2_O_2_ 30% *v*/*v*) in a 1:1 *v*/*v* ratio [7,24]. The Ti6Al4V specimens were immersed in the reaction mixture for 1 h. Subsequently, silanization was performed, involving the incorporation of functional organosilanes to facilitate protein-titanium bonding. The samples were immersed in a solution of 3-aminopropyltriethoxysilane (APTES) and toluene in a 1:20 *v*/*v* ratio at 100 °C for two different durations, 3 h and 14 h [9]. After each stage, the samples were washed as described in Section 2.1.

### 3.3. Microalgal Biomass

Biofunctionalization was conducted using wet microalgal biomass with high protein content. The strains used were *Chlorella sorokiniana* UTEX 1230 and *Synechococcus* sp. PCC 7002, obtained from UTEX (The Culture Collection of Algae the University of Texas at Austin, TX, USA) and cultivated in 20-L photobioreactors under natural photoperiods. Various nitrogen concentrations in the culture medium were evaluated to maximize the protein content in the biomass [22]. Four levels were assessed, resulting in a total of 15 experimental units including the control. The media used were Basal Bristol Medium (BBM) for strain 1230 and Zarrouk’s medium for 7002. The nitrogen concentrations evaluated for both media were 0.25 g/L (T1), organic fertilizer (17.2 g/L) (T2), synthetic fertilizer (0.5 g/L) (T3), and BBM medium (0.90 g/L) (T4). The protein content of the biomass from each treatment was quantified using the Kjeldahl method, and the best result was selected for subsequent bromatological analysis. 

### 3.4. Immobilization

Titanium samples were immersed in solutions of the selected biomass from Section 2.3 and deionized water at different concentrations (3 g/L and 5 g/L) and immersion times (3 h and 14 h) [3] with constant agitation at 80 rpm. Post-immersion, the samples were rinsed with 90% *v*/*v* ethanol.

### 3.5. Surface Characterization

#### 3.5.1. Contact Angle Measurement 

To assess the effectiveness of the activation process, the contact angle was determined using the sessile drop technique with a goniometer (Bonitech SDC-100, Jinan Boni Technology Co., Ltd., Jinan, Shandong, China) [16]. A 2-µL drop of bidistilled water was deposited on the surface of the specimen. A photograph of the drop was taken using a 1.6X objective, and the images were analyzed with ImageJ version 1.54k. 

#### 3.5.2. Scanning Electron Microscopy (SEM)

The chemical modifications during each stage of the biofunctionalization process were analyzed using Energy Dispersive X-ray Spectroscopy (EDS) (ZEISS EVO 10, Zeiss, Oberkochen, Germany) to identify chemical elements or organic compounds present on the various surfaces. Morphological characterization of all samples, including polished, activated, silanized, and biofunctionalized surfaces, was conducted using scanning electron microscopy (SEM) ZEISS EVO 10. The range of the operating voltage was between 15 and 20 kV. The samples were affixed using carbon tape and coated with an Au-Pd alloy with a thickness in the presence of Argon gas.

### 3.6. Cell Assays

#### 3.6.1. Viability and Cytotoxicity

The cytotoxicity of the materials on Saos-2 cells was determined using the MTT assay (3-(4,5-dimethylthiazol-2-yl)-2,5-diphenyltetrazolium bromide). Cells were seeded onto the sample surfaces in McCoy’s medium with 10% fetal bovine serum. The samples were incubated for 48 h at 37 °C with 5% CO_2_. Following incubation, MTT was added, and after 4 h at 37 °C, DMSO was introduced. Absorbance at 570 nm was measured using a spectrophotometer (Thermo Scientific Varioskan LUX Multimode Microplate Reader, Thermo Fisher Scientific, Waltham, MA, USA). The assays were performed in two independent experiments, each in duplicate.

#### 3.6.2. Cell Adhesion

Cells were cultured on the samples in McCoy’s medium for 24 h at 37 °C and 5% CO_2_. After incubation, a fixation process was conducted for observation via scanning electron microscopy (JEOL JMS6490LV, Tokyo, Japan).

### 3.7. Statistical Analysis

Statistical analysis of the results was performed using analysis of variance (ANOVA) with a 95% confidence level, employing STATGRAPHICS Centurion version XVI. Tukey and multiple range tests were used to establish which treatments had effects on the response variables. The results are reported as mean values with standard errors.

## 4. Conclusions

The modification of the culture medium for *Chlorella sorokiniana* UTEX 1230 resulted in a significant increase in the protein content of the biomass. In contrast, this modification did not enhance the protein content in *Synechococcus* sp. PCC 7002. Furthermore, hydrophilicity increased with activation using the piranha solution, as confirmed by contact angle measurements, where the angle decreased from an initial 76° to 18° post-treatment. The silanization process was effective in inducing both physical and chemical modifications on the activated surfaces, confirming the formation of the silane layer. This process yielded more favorable results in samples treated for 14 h.

Regarding biomass immobilization, it was observed that higher concentrations and prolonged exposure times increased the amount of biomass on the material surface for both microalgae. However, it was found that particle size plays a crucial role in surface functionalization, with the smaller microalga, in this case, strain 7002, adhering more effectively to the surface. Consequently, cellular assays were conducted using this sample, and it was determined that the treatments did not affect cell viability compared to control cells. Therefore, these treatments could potentially be classified as non-cytotoxic, with a viability value of 86.8%. Additionally, SEM microscopy revealed a considerable number of cells adhered to the treated sample, displaying a polyhedral morphology characteristic of this cell type and the formation of a cellular monolayer. These findings suggest that this type of coating could be a potential strategy to enhance osseointegration.

## Figures and Tables

**Table 1:** Protein content for 1230 and 7002.

Treatment	Protein (%)	
	Strain 1230	Strain 7002
T1	17.17	48.1
T2	11.17	25.6
T3	47.70	11.7
T4	38.70	15.4

**Table 2:** Proximate analysis of the obtained biomass.

Parameter (%)	Strain	
	1230	7002
Humidity	8.19	9.52
Ashes	16.86	18.81
Proteins	47.70	48.10
Lipids	11.13	13.57
Fiber	0.54	0.41
Carbohydrates	15.58	9.61

**Table 3:** EDS results of the titanium surface, and surfaces silanized for 3 h and 14 h.

Atom	Control		Sil 3 h		Sil 14 h	
	Weight %	Atomic %	Weight %	Atomic %	Weight %	Atomic %
Ti	88.97	84.70	78.55	63.52	79.17	58.06
Al	6.82	84.70	4.20	5.65	1.70	2.22
V	4.22	3.77	3.77	5.89	4.52	3.12
C	0	0	10.97	25.84	6.97	20.26
Si	0	0	0.33	0.44	0.54	0.83

**Table 4:** Elements present in the EDS spectra of 1230.

Time	3 h				14 h			
Atom	3 g/L		5 g/L		3 g/L		5 g/L	
	Weight %	Atomic %	Weight %	Atomic %	Weight %	Atomic %	Weight %	Atomic %
Ti	43.11	35.26	43.29	34.82	46.91	42.01	53.23	44.22
Al	2.78	3.10	2.06	2.95	2.36	3.75	3.36	4.78
V	3.15	2.57	3.32	2.53	3.16	2.66	3.25	2.60
Si	0.43	0.56	0.32	0.44	0.54	0.83	0.43	0.59
C	3.45	10.39	3.39	10.86	3.23	11.55	3.94	12.18
O	15.67	35.32	16.99	40.92	11.24	30.14	12.05	27.94
N	2.10	3.87	0.81	2.23	2.03	6.10	2.15	7.51
P	1.45	1.35	1.15	1.41	1.11	1.43	1.20	1.53

**Table 5:** The elements present in the EDS spectra of strain 7002.

Time	3 h				14 h			
Atom	3 g/L		5 g/L		3 g/L		5 g/L	
	Weight %	Atomic %	Weight %	Atomic %	Weight %	Atomic %	Weight %	Atomic %
Ti	36.31	28.50	48.80	37.62	42.33	52.23	45.40	32.75
Al	2.28	3.18	2.70	3.70	3.45	3.45	2.63	3.37
V	3.12	2.87	3.34	2.42	2.56	3.45	2.69	1.82
Si	0.41	0.65	0.34	0.55	0.57	0.84	0.44	0.60
C	4.27	13.36	6.12	16.21	4.55	13.78	4.94	14.23
O	17.34	40.76	13.13	30.30	12.05	30.12	15.29	36.23
N	2.57	6.05	3.53	9.32	2.15	6.13	2.54	6.27
P	1.35	1.45	1.05	1.51	1.23	1.53	1.20	1.63

**Table 6:** The effect of materials on the viability of Saos-2 cells.

Treatment	% Viability Saos-2 X ± DS
Control Ti	88.9 ± 6.8 ^a^
7002	86.8 ± 8.3 ^a^
Untreated control	100.0 ± 4.1 ^b^

The superscript letter indicates a significant difference (*p* < 0.05) within the same column.

## Data Availability

The data presented in this study are available on reasonable request from the corresponding author.
